# Toxic Effects of Di-2-ethylhexyl Phthalate: An Overview

**DOI:** 10.1155/2018/1750368

**Published:** 2018-02-22

**Authors:** Sai Sandeep Singh Rowdhwal, Jiaxiang Chen

**Affiliations:** ^1^Department of Physiology, Medical College of Nanchang University, Nanchang 330006, China; ^2^International Exchange College of Nanchang University, Nanchang 330006, China; ^3^Jiangxi Provincial Key Laboratory of Reproductive Physiology and Pathology, Medical College of Nanchang University, Nanchang 330006, China

## Abstract

Di-2-ethylhexyl phthalate (DEHP) is extensively used as a plasticizer in many products, especially medical devices, furniture materials, cosmetics, and personal care products. DEHP is noncovalently bound to plastics, and therefore, it will leach out of these products after repeated use, heating, and/or cleaning of the products. Due to the overuse of DEHP in many products, it enters and pollutes the environment through release from industrial settings and plastic waste disposal sites. DEHP can enter the body through inhalation, ingestion, and dermal contact on a daily basis, which has raised some concerns about its safety and its potential effects on human health. The main aim of this review is to give an overview of the endocrine, testicular, ovarian, neural, hepatotoxic, and cardiotoxic effects of DEHP on animal models and humans* in vitro *and* in vivo*.

## 1. Introduction

Di-2-ethylhexyl phthalate (DEHP) is the most common member of the class of phthalates, which are used as plasticizers in polymer products to make plastic flexible. DEHP is also called bis(2-ethylhexyl) phthalate or dioctyl phthalate (DOP). This colorless viscous and lipophilic liquid is more soluble in materials such as paint removers, gasoline, and oils than in water and has almost no odor [1]. It does not evaporate easily, and little will be present in the air even near sources of production. DEHP is produced at more than 2 million tons annually worldwide [2].

DEHP consists of a pair of eight-carbon esters linked to a benzene-dicarboxylic acid ring with a molecular weight (MW) equal to 390.56 g-mol^−1^ and a chemical formula of C_24_H_38_O_4_. DEHP is used as a plasticizer in many products, especially in medical devices, such as intravenous (IV) bags and tubing, umbilical artery catheters, blood bags and infusion tubing, enteral nutrition feeding bags, nasogastric tubes, and peritoneal dialysis bags, and is utilized in manufacturing a wide variety of consumer products, such as packed food and beverages; soft plastic products, such as toys and infant products [3], building and furniture materials, including furniture upholstery, mattresses, wall coverings, floor tiles, and vinyl flooring; and cosmetics and personal care products to carry fragrances [4, 5]. Due to the overuse of DEHP in many products, it can be found in air, water, and soil. DEHP can bind to the dust particles in air and be carried back to earth when it is released; DEHP can also bind strongly to soil and dissolves very slowly in groundwater [6].

**Figure 1:**
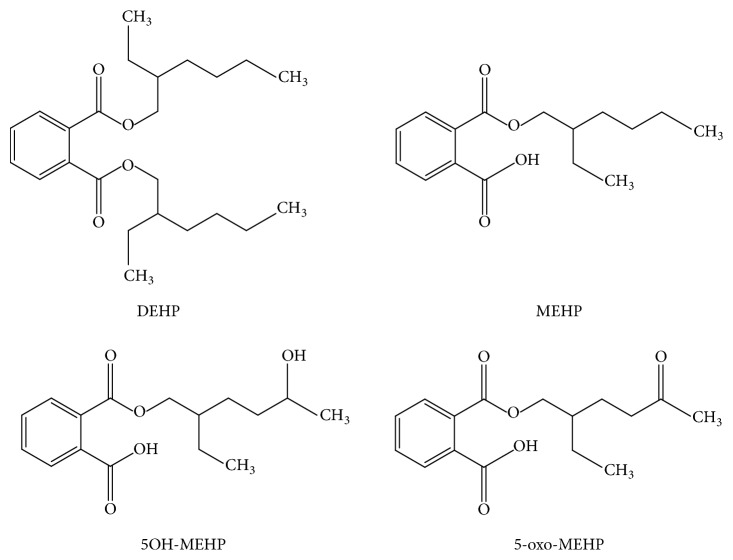
DEHP and its main metabolites.

Due to its ubiquity in the environment, DEHP has raised concerns pertaining to continuous exposure of the human population. When DEHP enters the body, it is metabolized into different metabolites. The primary monoester metabolites of DEHP are di-n-octyl phthalate (DnOP), di-n-butyl phthalate (DnBP), benzyl butyl phthalate (BBzP), and diethyl phthalate (DEP) [7]. The secondary oxidation DEHP metabolites are mono-(2-ethyl-5-hydroxyhexyl) phthalate (5OH-MEHP), mono-(2-ethyl-5-oxohexyl) phthalate (5-oxo-MEHP), mono-(2-ethyl-5-carboxypentyl) phthalate (5cx-MEPP), and mono-[2-(carboxymethyl) hexyl] phthalate (2cx-MMHP) (Figure 1) [8]. Human CYP2C9(*∗*)1 and CYP2C19 are the major CYP isoforms producing 5OH-MEHP and 5-oxo-MEHP metabolites, while only human CYP2C9(*∗*)1 and 2C9(*∗*)2 can produce 5cx-MEPP from MEHP [9].

Epidemiological studies showed that DEHP can be found in different products such as meat and lipid-rich products such as fats and dairy products at higher concentrations (≥300 *μ*g/kg) [10]. Recent studies reported that bread, as an important source of nutrients, contributes to the total exposure at rate of 31.4% in the general adult population [11]. According to the Environmental Protection Agency (EPA), the reference dose of 20 *μ*g/kg/day poses a risk of hepatomegaly, and the European Food Safety Authority (EFSA) reported that a dose of 50 *μ*g/kg/day can lead to testicular toxicity [10]. The results from an analysis of the exposure to DEHP from blood bags indicated that the maximum level of human exposure to DEHP released from blood bags is 0.7 mg/kg weight/time [12].

## 2. DEHP and Endocrine Toxicity

DEHP is best known as an endocrine disruptor (ED). An endocrine disrupter is an exogenous substance or mixture that alters the function(s) of the endocrine system and consequently causes adverse health effects in an intact organism, its progeny, or (sub)populations [13]. In utero DEHP exposure diminishes mineralocorticoid receptor (MR) expression in adult rat Leydig cells, which affects aldosterone-induced androgen formation, which probably decreases testosterone production [14]. Further investigations revealed that a decrease of 50% in aldosterone and testosterone concentrations in male rats was due to in utero exposure to DEHP at doses of 100, 300, and 750 mg kg^−1^ day^−1^ but corticosterone levels did not change [14, 15]. This could be explained by a significant decrease in adrenal tissue weight following 750 mg kg^−1^ day^−1^ DEHP exposure due to diminished angiotensin II (AT) receptor levels in adrenal tissue. Interestingly, components of the renin-angiotensin-aldosterone system (RAAS) and stimulants of aldosterone show no change in the serum [15].

DEHP is highly toxic, with an LC_50_ of 0.50 ppm, and leads to embryo mortality and typical toxicity symptoms, such as tail curvature, necrosis, cardiac edema, and no-touch response, in zebrafish. DEHP can enhance estrogenic activity at concentrations of 1.50 ppm* in vitro* and* in vivo*, suggesting that DEHP induces the transactivation of ER in an addictive manner [16].

DEHP decreases the expression of steroidogenic acute regulatory protein (StAR) mRNA in pregnant mice, which reduces steroidogenesis catastrophically in both mice and humans. DEHP also lowers the in utero fetal testicular mRNA levels of 17*α*-hydroxylase and cytochrome P450 17A1, which are key enzymes in the steroidogenic pathway [17]. The above two conditions can occur from either direct exposure of fetal testis or indirect maternal exposure.

Aldosterone can induce or activate MR in rat Leydig cells, which enhances testosterone production by an aldosterone-mediated MR mechanism [18]. Based on all the factors explained above, the reduced MR expression in Leydig cells [14] and decreased aldosterone serum levels [19], both provoked by DEHP in utero exposure, probably can reduce testosterone levels in adult rats.

After DEHP exposure, histopathological thyroid changes occurred: T_4_ levels but not T_3_ levels declined when rats were exposed to DEHP [20]. It has been observed that rats fed with a diet containing DEHP show consistent thyroid changes, with persistent hyperactivity due to increases in the number and size of lysosomes, hypertrophy of the Golgi apparatus, and dilation of the rough endoplasmic reticulum [21]. DEHP metabolites such as MEHP were associated with altered free T_4_ levels in adult men [22]. Conversely, rats intravenously given DEHP at concentrations representing the amount that can leach from PVC blood bags used for human blood transfusions had increased serum T_3_  and  T_4_ levels [23]. These studies indicate that the toxic effect of DEHP and its metabolites is different in different species and the toxic effect is related to the dose.

The potential for DEHP and its metabolites, such as MEHP, to disturb the thyroid endocrine system has been exposed by the experiments done on zebrafish larvae, proving that post-DEHP exposure not only alters the levels of thyroid hormones but also affects their synthesis, regulation, and action [24, 25]. Thyroid hormones are known to play an important role in growth, differentiation, and metabolism in early embryonic stages of zebrafish [26]. Expression of hypothalamic-pituitary-thyroid (HPT) axis-related genes is disrupted, which demonstrates the toxicity of DEHP on thyroid hormones in zebrafish larvae [24]. MEHP, a DEHP metabolite, has the same endocrine-disrupting effect on the thyroid by altering the series of gene transcription involved in the HPT axis [25].

Diester DEHP can be metabolized to the primary monoester MEHP* in vitro* in human thyroid cells. DEHP (10 *μ*M) and 100 *μ*M MEHP can induce thyroglobulin (Tg) and cAMP secretion; however,* in vitro* DEHP exposure does not alter the gene expression of thyroid-specific genes [27]. Studies on elderly people suggest that insulin resistance is increased after DEHP exposure due to an imbalance between oxidative stress production and antioxidant defenses, as DEHP is known to induce oxidative stress, which might also lead to insulin resistance in elderly people [28]. DEHP can disturb thyroid hormone homeostasis and reduce TH levels [triiodothyronine (T3), thyroxine (T4), and thyrotropin-releasing hormone (TRH) can be reduced] by activating the Ras/Akt/TRHr pathway and affect hepatic enzymes that play vital roles in thyroid-disrupting effects [29].

In Chinese school children, exposure to MEHP was positively associated with BMI and waist circumference, which are related to obesity. Mono-(2-ethyl-5-hydroxylhexyl) phthalate (MEHHP) and mono-(2-ethyl-5-oxohexyl) phthalate (MEOHP) were found to be significantly associated with BMI only in the 8–11-year age group, not in all the subjects. Because younger children experience more rapid growth, their growth is more likely to be affected by external disturbances [30]. A cross-sectional analysis of the NHANES data showed that MEHHP and MEOHP, DEHP metabolites, were associated with increased waist circumference and BMI in males. There were a positive relationship between MEHHP and BMI among 20–59-year-old males and a nonmonotonic increase among 12–19-year-old males [31]. DEHP has weak hormonal activities and may affect male fertility in rats when given at high oral doses. In conclusion, the evidence linking personal care products to endocrine-disruptive effects in humans is lacking for the most part.

## 3. DEHP and Testicular Toxicity

Administration of DEHP orally by gavage in rats and mice at higher doses leads to toxic effects in the testes. The potential effects of DEHP have been found to interfere with normal sexual development in male rodents (mice, rats, and guinea pigs) resulting from reduced testosterone synthesis (decreased sperm production) [32].

Testicular toxicity of DEHP at gonadotoxic levels reduces the litter size, which is associated with testicular atrophy, reduced epididymal sperm density and motility, and increased numbers of abnormal sperm in male rats [33]. However, the dose response to DEHP is not nonmonotonic during puberty. Low doses could accelerate puberty; however, low doses of 10 or 100 mg/kg/day did not lower the age at puberty or enhance testosterone levels, and high doses of DEHP (300 and 900 mg DEHP/kg/day) did not increase serum testosterone or accelerate puberty [34].

Zinc is a major part of seminal plasma, which originates from the prostate in the male reproductive system. It maintains testicular functions in the testis [35]. During the process of spermatogenesis, zinc will be incorporated in the germ cells, and there is a gradual increase in the zinc concentrations in the testis, which suggests that zinc helps in the maintenance and regulation of sperm motility and spermatogenesis [36]. Gonadotoxic concentrations of DEHP may lead to atrophy of seminiferous tubules, lowered sperm motility, and induced structural abnormalities in sperm because of the rapid loss of zinc from the spermatids [33]. DEHP-induced testicular atrophy at doses of 500 mg/kg or more appears to be caused by inhibition of DNA replication, activation of a response to DNA damage, SIRT1 attenuation, and accelerated cell death via induction of mitochondrion-associated intrinsic apoptosis. DEHP can induce apoptosis partially by stalling replication forks, leading to DNA strand breaks, the induction of mitochondrial damage, and increased reactive oxygen species (ROS) production. Activation of DNA damage leads to SIRT1 (regulator of mitochondrial function) attenuation and thereby suppresses testicular ATP levels. The DEHP-induced attenuation of ATP levels may be a crucial problem to male fertility since the motility of spermatozoa depends on sperm ATP [37].

Acute testicular toxicity of MEHP was investigated in 28-day-old male Wistar rats 3, 6, and 12 hours after a single oral dose (by gavage) of 400 mg/kg bw; detachment and sloughing of germ cells were reported [38]. Subchronic (13-week) feeding studies were conducted in F344 rats: testicular atrophy was observed in all male rats fed with 25,000 ppm DEHP and was present but less pronounced in rats fed with 12,500 ppm DEHP. Chronic (2-year) studies were also conducted in F344 rats and B6C3F_1_ mice. In male rats, pituitary hypertrophy and testicular atrophy were observed in the 12,000 ppm DEHP-treated group. In male mice, testicular degeneration was reported in the 6,000 ppm DEHP-treated group. However, no general toxicity was reported in female mice [39].

*In vivo* exposure of DEHP at a concentration of 500 mg/kg/day in mice led to a reduction in the fertilization ability of spermatozoa and embryos and a decline in developmental potentiality. Treatment of mice with DEHP for 1 month resulted in a 3-fold increased frequency of mutations in genomic DNA. DEHP also increased mutagenic risks, especially in the testis of mice [40]. Embryonic exposure to DEHP in mice disrupted testicular germ cell disorganization and impaired spermatogonial stem cells of the progeny of multiple generations (F1–F4) [41]. There was a 27% decrease in the sperm count of the progeny of F1 but a 7.8% decrease in the sperm count of F2–F4 progeny, which explains the 3-fold greater decreased sperm count when compared to F2–F4 [41].

First-trimester DEHP exposure in pregnant women has negative impacts on the offspring that lead to a decrease in the anogenital distance (AGD), particularly in newborn boys but not in girls, which implies that exposure to DEHP affects male genital development [42]. It has also been reported that first-trimester DEHP exposure is associated with newborn genital anomalies primarily driven by an isolated hydrocele, which make up the majority of anomalies in newborn males [43].

## 4. DEHP and Ovarian Toxicity

The ovaries are a significant primary reproductive organ that plays an important role in female gamete production and the release of sex hormones. Functional disturbances of ovaries can cause many reproductive problems such as anovulation, irregular estrogen secretion, and sterility [44]. DEHP targets the ovary potentially through its metabolite MEHP [45].

The antral follicle is an ovarian reserve and an important supplier of sex steroid hormones in females, and estradiol (E2) is necessary for follicle growth. DEHP affects follicle growth through a reduction of E2 levels* in vitro* [46]. A reduction of estradiol could decrease the expression of the* Arom* gene, which produces the aromatase enzyme, which converts testosterone to estradiol. Hence, decreased expression of the* Arom* gene can decrease serum E2 levels [47].

Usually, follicle function requires proper regulation of steroidogenesis and survival from atresia [48]. Atresia is a natural apoptotic occurrence by which follicles undergo death and has a harmful effect on ovarian and reproductive health. Ovarian follicular atresia is controlled by proapoptotic factors (such as Bad, Bax, and Bok) and antiapoptotic factors (such as Bcl2 and Bcl2l10), which are generally dysregulated by DEHP exposure [48]. Induction of oxidative stress by DEHP is another cause leading to follicular atresia [49]. DEHP can also inhibit follicle growth via oxidative stress [50].

Mice treated with DEHP at a dosage of 20 *μ*g/kg/day–750 mg/kg/day had altered estrous cyclicity and accelerated primordial follicle recruitment due to the dysregulation of ovarian mRNA and altered levels of proteins in the phosphatidylinositol 3-kinase (PI3K) signaling pathway, which is associated with early folliculogenesis. There was decrease in the percentage of primordial follicles after DEHP exposure. It is evident that low doses of DEHP can interfere with normal reproductive functions [51].

*In vitro *studies on neonatal ovaries from mice exposed to DEHP (0.2–20 *μ*g/ml) revealed that DEHP was metabolized to MEHP, causing a decrease in steroidogenic enzyme levels that led to a decrease in testosterone, estrone, and E2 levels. MEHP accelerated primordial follicle recruitment potentially via overactivation of ovarian PI3K signaling [45].

Oral administration of DEHP and its metabolite MEHP has been shown to have negative impacts on oocyte meiotic maturation and development* in vivo*. The reduced developmental ability could be due to lowered expression levels of the* Pou5f1*,* Asah1*, and* Ccna1* genes. This suggests that DEHP-induced alterations in gene expression activity could explain how DEHP compromises fertility [52].

## 5. DEHP and Endometriosis

Endometriosis is a benign gynecological problem affecting more than 10% of women of reproductive age and is defined as a disease in which tissue that normally grows inside the uterus grows outside it [53, 54]. DEHP is known as an environmental contaminant with potentially adverse effects on the fertility of animals, and evidence has shown that environmental toxins may directly or indirectly affect the response of the endometrium to steroids and even lead to endometriosis [55].

A study on Indian women with endometriosis showed significantly higher levels of DEHP exposure compared with women without endometriosis [56]. Women with advanced-stage endometriosis in a Korean population had higher plasma concentrations of DEHP and its metabolites such as MEHP, which supports the hypothesis that exposure to phthalates might play a role in the pathogenesis of endometriosis [57].

Many studies have suggested that DEHP can cause hormonally mediated diseases such as endometriosis in reproductive-age women [56, 58]. Cobellis et al. showed that the plasma DEHP concentration was associated with endometriosis, suggesting that DEHP plays a potential role in the establishment of endometriosis [59]. DEHP treatment of endometrial cells* in vitro *has been shown to increase ROS, which indicates that DEHP can induce oxidative stress in human endometrial cells [60] and DEHP-associated endometrial stromal cell alterations may be associated with the progression of the pathogenesis of endometriosis [61].* In vitro* exposure to DEHP can lead to the establishment of endometriosis by increasing the invasive and proliferative activities of endometrial cells because DEHP can increase metalloproteinase- (MMP-) 2 and MMP-9 activities and cellular invasiveness.

## 6. DEHP and Renal Toxicity

DEHP is reported to be nephrotoxic in mice, and DEHP may exacerbate this pathological change and lead to chronic progressive nephropathy. Studies have shown that chronic progressive nephropathy was observed in male mice treated with 1500 ppm DEHP. After 104 weeks of DEHP exposure, kidney weights were significantly lower than those of the controls [62]. PPAR-alpha was shown to mediate the subacute-chronic toxicity of DEHP in kidneys [63]. DEHP can also cause nephropathy in male rats. DEHP exposure exacerbated age-, species-, or strain-related lesions such as mineralization of the renal papilla and chronic progressive nephropathy. Renal tubule pigmentation was seen in male and female rats at a dosage of 12,500 ppm [64]. It was suggested that DEHP-induced nephropathy in rodents may be related to peroxisome proliferation, which has been observed in the renal proximal tubules [65]. However, DEHP had no effect on the kidneys of male cynomolgus monkeys treated with 500 mg/kg/day DEHP for 14 days [66].

The highest levels of measurable DEHP were detected in the kidneys of rats and could cause a significantly higher incidence of focal cysts and a significant decrease in kidney function as demonstrated by creatinine clearance [67]. Patients receiving long-term dialysis may acquire PKD secondarily from their exposure to chemicals leached from artificial kidneys [67]. Assessment of the degree of exposure to DEHP in 21 patients with chronic renal failure undergoing maintenance hemodialysis was carried out using plasticized tubing. The plasma level of DEHP increased. The total amount of DEHP retained by the patient during the dialysis session ranged from 3.6 to 59.6 mg, which suggests that patients on hemodialysis are always regularly exposed to considerable amounts of DEHP [68].

However, patients undergoing hemodialysis using tubing plasticized with only DEHP are regularly exposed to nonnegligible amounts of DEHP. In view of several biological effects previously reported, it is time to reconsider the use of only DEHP as a plasticizer. Highly unacceptable amounts of DEHP leached during dialysis session could be easily avoided by careful selection of hemodialysis tubing.

## 7. DEHP and Neurotoxicity

The brain has been determined to be at risk of DEHP exposure. DEHP can affect neurodevelopment and lead to teratogenic anomalies by disrupting normal fetal brain development, as DEHP can cross the placenta and enter the fetal circulation [69, 70]. Gestational and postnatal DEHP exposure has harmful effects on rat brain development and function [69]. DEHP exposure (1500 mg/kg) in utero led to a metabolic disturbance of the lipid metabolome of the fetal rat brain, which caused anomalous brain growth [70].

The hippocampus is a major part of the brain that plays an important role in memory and spatial navigation. Post-DEHP exposure in postnatal rats has been shown to have a harmful impact on the development of the hippocampus in males but not in females [71]. Resistance of the female rat hippocampus to modification by DEHP is because DEHP alters the lipid profile of the hippocampus during postnatal development, leading to elevated levels of phosphatidylcholine and sphingomyelin in the hippocampus of female rats but no effect of DEHP on the abundance of phosphatidylcholine and sphingomyelin in the hippocampus of male rats. These studies suggested that upregulation of hippocampal lipids may serve a neuroprotective role in DEHP-exposed female rats [72].

Brain-derived neurotrophic factor (BDNF) is a protein that plays a critical role in the survival of existing neurons and promotes the enhancement and differentiation of new neurons and their synapses [73]. Low-dose DEHP exposure (10 mg/kg) has been shown to affect dorsal hippocampal BDNF expression, which is downregulated in male rats. DEHP-exposed male rats have been observed to have decreased dendritic spine density [71]. As BDNF is important for dendritic growth and makes synaptic connections between neurons, there could be an underlying mechanism decreasing BDNF expression, which could decrease dendritic spine density directly or indirectly [73].

In addition to rats, the neurotoxicity of DEHP has also been observed in* Caenorhabditis elegans*, a nematode. DEHP exposure can lead to an accumulation of ROS intracellularly, which causes neurotoxicity. DEHP exposure can also inhibit the expression of many genes necessary for differentiation and function of AFD sensory neurons (necessary for the thermosensory response) [74].

Studies on the effect of perinatal DEHP exposure on the emotional behavior of mice have shown that phosphorylation of ERK1/2 in the hippocampus of pubertal mice and adult males is inhibited and that downregulation of androgen receptors in the pubertal male hippocampus and estrogen receptor (ER) *β* in pubertal females and the adult hippocampus of both sexes may be associated with anxiety- and depression-like behaviors in mice [75]. Intrauterine and lactational exposure to DEHP at low concentrations of 50 and 200 mg/kg/d decreased the levels of the N-methyl-d-aspartic acid (NMDA) receptor subunits NR1 and NR2B in the hippocampus in offspring mice, leading to impaired spatial learning and memory [76].

In the 104-week feeding studies mentioned above, DEHP caused an increase in the mean relative brain weights in male B6C3F1 mice at the highest dose (6,000 ppm). A similar effect was observed in male and female F344 rats at the highest dose of 12,500 ppm [62, 64].

## 8. DEHP and Hepatotoxicity

Continuous oral exposure to DEHP of rodents can cause hepatomegaly, which is due to hyperplasia and hypertrophy of liver parenchymal cells. Microscopic observations revealed a decrease in glycogen stores, a periportal accumulation of fat, and an accumulation of lipofuscin granules [77]. The modes of action of DEHP in hepatocytes include (1) activation of PPAR*α*, (2) proliferation of peroxisomes and induction of peroxisomal proteins, (3) induction of nonperoxisomal metabolism proteins, (4) induction of cell proliferation, (5) suppression of apoptosis, (6) production of reactive oxygen species, (7) oxidative DNA damage, and (8) inhibition of gap junctional intercellular communication [78].

*In vitro* and* in vivo* experiments explained that DEHP-stimulated activation of peroxisome proliferator-activated receptor *γ* (PPAR*γ*) leads to the production of oxidative stress and downregulated expression of insulin receptor and GLUT4 proteins, disrupting the insulin signaling pathway in the liver of SD rats and L02 cells [79].

Peroxisome proliferation results in an elevation of fatty acid metabolism, which is a characteristic response to DEHP exposure in the liver in rodents [80]. It has been believed that peroxisome proliferation and the accumulation of lipofuscin granules are related to hepatocarcinogenesis after DEHP exposure [81]. Events such as the induction of cell proliferation, decreased apoptosis, and oxidative DNA damage have also been proposed to be significantly involved in DEHP-induced hepatocarcinogenesis [82]. According to evidence from rodent studies, the International Agency for Research on Cancer (IARC) has classified DEHP as a carcinogenic 2B substance [83].

In rodents, DEHP-induced liver carcinogenesis involves multiple pathways contributing to tumor formation: (1) rapid metabolism of the parental compound to primary and secondary bioactive metabolites that are readily absorbed and distributed throughout the body, (2) receptor-independent activation of hepatic macrophages and production of oxidants, (3) activation of PPAR*α* in hepatocytes and sustained increases in the expression of peroxisomal and nonperoxisomal metabolism-related genes, (4) enlargement of many hepatocellular organelles (peroxisomes, mitochondria, etc.), (5) rapid but transient increases in cell proliferation and decreases in apoptosis, (6) sustained hepatomegaly, (7) chronic low-level oxidative stress and accumulation of DNA damage, (8) selective clonal expansion of initiated cells, (9) appearance of preneoplastic nodules, and (10) development of adenomas and carcinomas [84].

DEHP can induce oxidative stress by disturbing the antioxidant balance in hepatocytes. The selenium (Se) status plays a crucial role in protecting the liver structure and function because it is a redox regulator. DEHP exposure can lead to Se deficiency and an increase in oxidative stress in rat hepatocytes [85]. Induced oxidative stress and DNA damage, which activated the p53-dependent apoptotic pathway* in vivo* and* in vitro*, were observed after DEHP exposure in hepatocytes [85, 86]. Mdm2, an important negative regulator of p53, was also suppressed by DEHP. Therefore, DEHP induced p53-dependent apoptosis via the induction of oxidative stress [86].

DEHP-exposed rats have been shown to have increased body weight and increased levels of accumulated triglycerides due to an oppositely regulated activation of the JAK/STAT pathway in liver and adipose tissue, leading to obesity because of abnormal body lipid metabolism [87]. DEHP can promote the accumulation of lipids by activating the SREBP-1c and PPAR*α* signaling pathway in hepatocytes* in vitro *[88]. However, no studies have shown that DEHP can activate PPAR*α* in human liver tissue or human hepatocytes* in vitro*. No studies have evaluated the potential for DEHP to cause cancer in humans, while exposure to high doses of DEHP for a long period of time resulted in liver cancer in rats and mice.

In most animal studies, the exposure amounts to DEHP of the animals were much higher than that in the environment. Furthermore, there are differences between animals and humans; it is difficult to predict some of the health effects of DEHP in humans by using these animal models.

## 9. DEHP and Cardiotoxicity

It has been identified that DEHP can be spread to heart tissue. Hillman et al. measured DEHP in neonatal hearts of individuals who had undergone umbilical catheterization and who had been supplied with blood products by gas chromatography-mass spectrometry and found significantly high levels of DEHP in heart tissue [89]. A more extensive distribution of DEHP was associated with a more widespread use of catheters made of DEHP. Lactational exposure to DEHP disrupted glucose oxidation in the cardiac muscles of F1 female albino rats [90]. In utero exposure to DEHP at a concentration of 300 mg/kg/day was followed by a decrease in the systolic and diastolic blood pressure in male offspring [19]. DEHP exposure results in metabolic remodeling of rat cardiomyocytes, which causes cardiac cells to increase their dependence on fatty acids (FA) for energy production, which results from the upregulation of genes associated with esterification, *β*-oxidation, fatty acid transport, and mitochondrial import and finally leads to ischemic injury or ventricular dysfunction of the heart [91].

DEHP can also decrease the conduction velocity of cardiomyocytes and asynchronous cell beating, which clearly explains the diminishing cardiac mechanical and electrical activity [92]. The mechanism behind the decrease in conduction velocity and asynchronous cell beating is due to a loss of gap junctional connexin-43 [92].* In vitro* DEHP exposure at a concentration of 250 *μ*M led to a change in cardiac electrical conduction, such as a decrease in heart rate and prolongation of PR and QT intervals. Acute cardiac effects with increasing doses of MEHP were studied in anaesthetized rats injected via the femoral artery. There were a steady and significant decrease in heart rate beginning after a total dose > 20 mg and a decline in blood pressure after a total dose > 40 mg [93].

After DEHP exposure, cardiomyocytes experienced a decrease in glucose oxidation and an increase in oxygen consumption, extracellular acidosis, mitochondrial mass, PPAR*α* expression, and myocyte fatty acid-substrate utilization [94]. DEHP had negative chronotropic and inotropic effects on human stem cell-derived cardiomyocytes [95] and reduced the intercellular connectivity of cardiomyocytes due to a loss of gap junctional connexin-43 [92]. DEHP exposure at appropriate levels reduced the transient amplitude of calcium, shortened the transient duration of calcium, decreased the decay time constant, and totally reduced the spontaneous beating rate [95].

## 10. Alternatives to DEHP

DEHP is very toxic and hazardous to organism health but is being widely used in our daily lives. DEHP exposure has been shown to have many adverse effects not only on animals but also on humans. DEHP is observed in the blood samples and urine samples of people who are hospitalized more because of the use of medical devices. Even rural communities away from hospitals are exposed in the environment through air or water or by packaged instant food products, which all can increase the exposure to DEHP. Kim conducted a risk assessment of DEHP in a work environment and suggested that the working environment should be managed to minimize the exposure to below 5 mg/m^3^ to protect the health of occupational workers [83].

DEHP exposure can be avoided by using PVC-free and DEHP-free alternatives. Using PVC-free products eliminates the concern over DEHP exposure because alternative polymers do not contain phthalates or any other softeners. These alternative polymers are naturally flexible and thus do not require a softening agent. Among these alternative materials, medical device manufacturers regularly use polyethylene, polypropylene, polyurethane and other polyolefins, silicone, ethylene vinyl acetate, and multilayer laminate plastics. Thus, hospitals have the option of choosing PVC-free medical devices. Increased efforts to develop viable replacement compounds, which necessarily include rigorous leaching, toxicity, and impact assessment studies, are needed before alternative plasticizers can be adopted as viable replacements.

Taken together, it is necessary to replace the DEHP products with DEHP-free plasticizers or PVC-free polymers in the future. However, any DEHP-free alternative should be thoroughly evaluated based on comprehensive toxicological studies, monitoring for long-term health effects, its functional effectiveness, and its cost efficiency.
